# 

**Published:** 1908-08-22

**Authors:** 


Telegraphic Address: Ospedale, London.

THE MODERN MEDICAL JOURNAL.

Telephone Number: 2734 Gerrard.

NEW SERIES. No. 76, Vol. III. No. 1148. Vol. XLIV. — SATURDAY, AUGUST 22ND, 1908. — PRICE THREEPENCE

